# β-Arrestin 1’s Interaction with TC45 Attenuates Stat signaling by dephosphorylating Stat to inhibit antimicrobial peptide expression

**DOI:** 10.1038/srep35808

**Published:** 2016-10-26

**Authors:** Jie-Jie Sun, Hui-Ting Yang, Guo-Juan Niu, Xiao-Wu Feng, Jiang-Feng Lan, Xiao-Fan Zhao, Jin-Xing Wang

**Affiliations:** 1 Shandong Provincial Key Laboratory of Animal Cells and Developmental Biology, School of Life Sciences, Shandong University, Jinan, Shandong, 250100, China

## Abstract

Impaired phosphatase activity leads to the persistent activation of signal transducers and activators of transcription (Stat). In mammals, Stat family members are often phosphorylated or dephosphorylated by the same enzymes. To date, only one Stat similar to mammalian Stat5a/b has been found in crustaceans and there have been few studies in Stat signal regulation in crustaceans. Here, we report that β-arrestin1 interacts with TC45 (45-kDa form of T cell protein tyrosine phosphatase) in the nucleus to attenuate Stat signaling by promoting dephosphorylation of Stat. Initially, we showed that Stat translocates into the nucleus to induce antimicrobial peptide (AMP) expression after bacterial infection. βArr1 enters the nucleus of hemocytes and recruits TC45 to form the βarr1-TC45-Stat complex, which dephosphorylates Stat efficiently. The interaction of TC45 with Stat decreased and Stat phosphorylation increased in *βarr1*-silenced shrimp (*Marsupenaeus japonicus)* after challenge with *Vibrio anguillarum*. βArr1 directly interacts with Stat in nucleus and accelerates Stat dephosphorylation by recruiting TC45 after *V. anguillarum challenge*. Further study showed that βarr1 and TC45 also affect AMP expression, which is regulated by Stat. Therefore, βarr1 and TC45 are involved in the anti-*V. anguillarum* immune response by regulating Stat activity negatively to decrease AMP expression in shrimp.

The Janus kinase (Jak)/signal transducers and activators of transcription (Stat) signaling pathway participates in cell proliferation, differentiation, development, survival and apoptosis^1^,^2^,^3^. Mammals, have Stats (Stat1, Stat2, Stat3, Stat4, Stat5a, Stat5b, Stat6), which are activated by tyrosine phosphorylation and translocated into the nucleus, where they bind to promoters to initiate transcription of effector genes^4^,^5^. Activators of Stats include epidermal growth factor receptor (EGFR)^6^,^7^, Jak^8^, phosphatidylinositol 3-kinase (PIK3)^9^, and tyrosine-protein kinase Src^10^. Inhibitors include the suppressor of cytokine signaling (Socs) family^3^,^11^,^12^, T cell protein tyrosine phosphatase (TC-PTP)^13^,^14^, receptor protein tyrosine phosphatase (RPTP)^15^, PTPN11/SHP2^16^, PTP-Meg2^17^, the protein inhibitor of activated Stat (PIAS) family^18^,^19^, and SIPAR (Stat3-interacting protein as a repressor)^20^. Protein tyrosine phosphatases (PTPs) are crucial negative regulators of Stat^16^,^17^. PTPs regulate Stat activity negatively either through dephosphorylation of protein tyrosine kinase Jak in the cytoplasm, e.g. PTPTC48^21^, or through direct dephosphorylation of Stat in the nucleus, e.g. by TC45^14^. Each PTP is capable of dephosphorylating multiple protein substrates. For example, TC45 mediates the dephosphorylation of Stat family members in the nucleus, such as Stat1, Stat3 and Stat5^14^,^22^,^23^.

In *Drosophila*, homologs of the mammalian Jak/Stat pathway components have been isolated, including ligands (Upds) encoded by the *unpaired* (*upd/os*) genes^24^, Domeless (Dome; also known as Mom)^25^, a Jak encoded by the *Hopscotch* (*hop*) gene^26^ and a Stat (also known as *Stat92E*), encoded by *marelle* (*mrl*)^27^,^28^. Several negative regulators of Jak/Stat pathway are also found, such as suppressor of cytokine signaling at 36E (Socs36E)^29^, ken and barbie (ken)^30^, protein inhibitors of activated stats (PIAS)^31^, and nucleosome remodeling factor (NURF)^32^. In invertebrates, if protein tyrosine phosphatases participate in regulating Jak/Stat pathway is still not clear.

β-Arrestins (βarrs) are multifunctional scaffold proteins that are involved in regulating desensitization and endocytosis of diverse cell surface receptors, such as G protein-coupled receptors^33^,^34^. Recent studies indicated that nuclear βarr1 directly interacts with Stat1 in the nucleus after IFN-gamma treatment and accelerates Stat1 dephosphorylation by recruiting TC45 (the 45-kDa form of TC-PTP)^35^. The C-terminal region of the TC45 has a specific bipartite nuclear localization sequence that targets the enzyme to the nucleus^36^. Another study also found that GdX (X-linked gene in the G6PD cluster at Xq28, also known as Ubl4A, Ubiquitin-like protein 4A) promotes Stat3 dephosphorylation by mediating the interaction between TC45 and Stat3 specifically^37^. In shrimp, only one Stat similar to mammalian Stat5a/b^38^,^39^ and two βarrs (βarr 1 and 2)^40^ have been identified, but TC45 has not been reported. Furthermore, the functions of βarrs and TC45 in the Jak/Stat pathway remain unknown.

In this study, we showed that after bacterial infection, Stat phosphorylation increased, followed by its translocation into the nucleus to induce AMP expression in shrimp hemocytes. βArr1, but not βarr 2, and TC45 accelerate the dephosphorylation of Stat in shrimp challenged by *V. anguillarum*. Further study showed that βarr1 recruits TC45 to phosphorylated Stat in the nucleus, and then mediates Stat dephosphorylation, leading to significantly decreased AMP expression in shrimp. Collectively, these results demonstrated that βarr1 acts as a scaffold protein to bridge the association between TC45 and Stat, which accelerates Stat dephosphorylation in the nucleus to moderate AMP expression in shrimp infected by *V. anguillarum*.

## Results

### Distributions of βarrs, TC45 and Stat in shrimp

**Figure 1:**
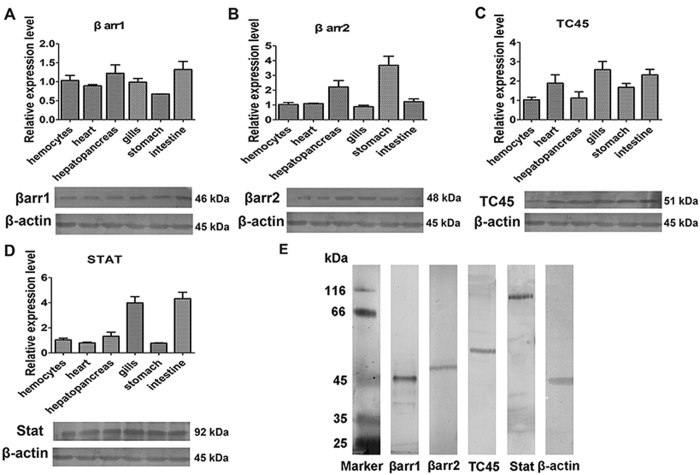
Tissue distributions of βarr1, βarr2 and TC45 and Stat in shrimp. (**A**–**D**) Tissue distributions of βarr1 (**A**), βarr2 (**B**), TC45 (**C**) and Stat (**D**) in shrimp, as analyzed by qRT-PCR (Upper panel) and western blotting (bottom panel). β-Actin was used as the control. The mRNA and proteins were extracted from hemocytes, heart, hepatopancreas, gills, stomach, and intestine and used for reverse transcription and western blotting. (**E**) Western blotting was used to indicate the molecular masses of native βarr1, βarr2, TC45, Stat and β-actin by detection using the corresponding polyclonal antibodies. The molecular weight markers were from Thermo Fisher Scientific, Lithuania. The experiments were repeats three times.

βarrs and Stat were reported in shrimp previously^40^,^41^. TC45 was first identified in shrimp and submitted to GenBank (no. KX358404). TC45 (51.06 kDa) contains a catalytic domain (22–292aa) and a nuclear localization signal at its non-catalytic C-terminal domain (293–451aa) with a sequence R × RKR, which is similar to TC45 in mammals^36^. QRT-PCR and western blotting were used to analyze the distributions of *βarr1*, *βarr2*, *TC45* and *Stat* transcripts and proteins. The results revealed that *βarr1* was expressed in hemocytes, heart, hepatopancreas, gills, stomach and intestine (Fig. 1A). *β Arr2* was mainly expressed in the hepatopancreas and stomach (Fig. 1B). *TC45* was expressed in hemocytes and other all tested organs (Fig. 1C). *Stat* was expressed in all tested organs, but was expressed at a relatively low level in hemocytes (Fig. 1D). Molecular masses of native βarr1, βarr2, TC45, Stat and β-actin were confirmed by western blotting using their corresponding antibodies (Fig. 1E). These results suggested that *βarr1*, *βarr2*, *TC45* and *Stat* are distributed ubiquitously in shrimp.

### Stat is involved in antibacterial immunity

**Figure 2:**
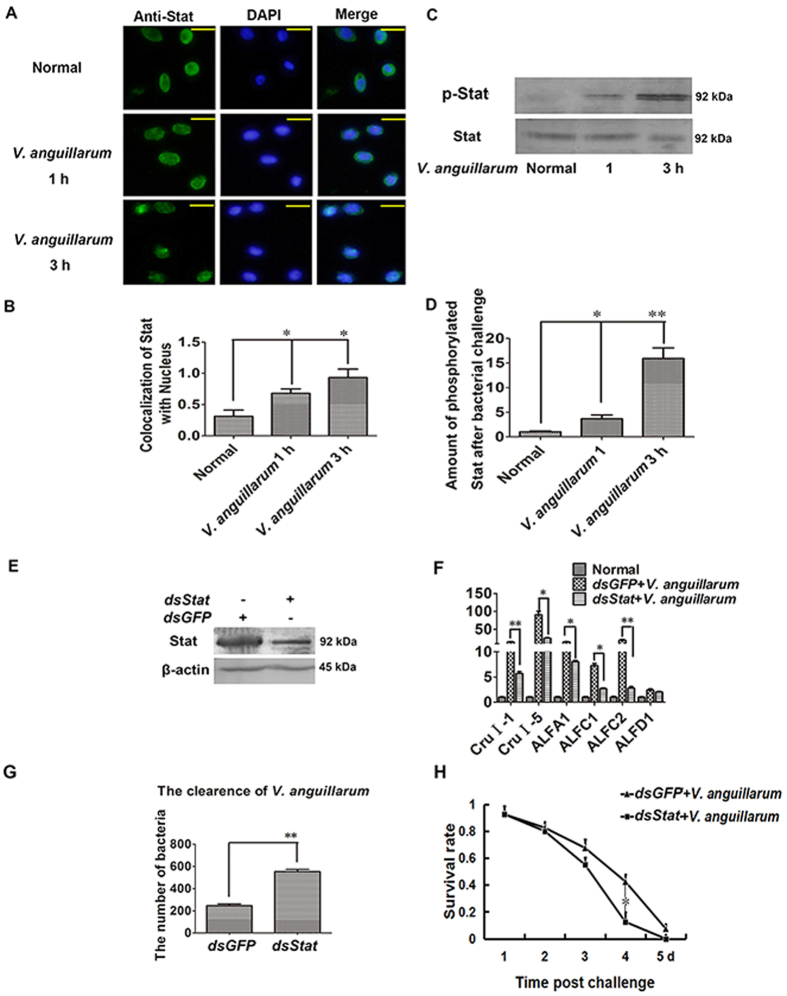
Stat translocates into the nucleus to induce AMP expression against bacterial infection. (**A**,**B**) Stat translocation in shrimp hemocytes challenged by *V. anguillarum* at 1 and 3 h was detected by immunocytochemistry using an anti-Stat antibody. (**B**) is the statistical analysis for the colocalization of Stat signal within the nucleus, as analyzed by WCIF ImageJ. (**C**,**D**) Stat phosphorylation in shrimp hemocytes challenged by *V. anguillarum* at 1 and 3 h, as detected by western blotting with an antibody recognizing phosphorylated Stat. (**D**) is the statistical analysis of C after digitization using Quantity One. (**E**) Western blotting was used to detect the RNAi efficiency of *dsStat* injection. (**F**) qRT-PCR detection of the expressions of *CruI-1*, *CruI-5*, *ALF-A1*, *ALF-C1*, *ALF-C2* and *ALF-D1* in *Stat*-silenced shrimp challenged by *V. anguillarum* at 6 h. (**G**,**H**) The bacteria clearance and survival rate of *Stat-*silenced shrimp were analyzed. ds*GFP* injection was used as the control. The experiments were repeats three times. The significant differences between two groups were analyzed using one-way ANOVA, followed by Tukey’s multiple comparison tests (*p < 0.05, **p < 0.01).

To study the function of Stat, we firstly detected Stat translocation in hemocytes using immunocytochemistry, and found that Stat could translocated into the nucleus in shrimp hemocytes when challenged with *V. anguillarum* (Fig. 2A,B). The phosphorylation of Stat was detected using anti-p-Stat (Abcam USA), which showed that phosphorylation of Stat in hemocytes was induced after challenge with *V. anguillarum* (Fig. 2C,D). The expression of AMPs regulated by Stat was also detected in *Stat* silenced shrimp, which showed that the expression of *CruΙ-1*, *CruΙ-5*, *ALF-A1*, *ALF-C1* and *ALF-C2* was not induced in *Stat*-silenced shrimp (Fig. 2E,F). To further confirm whether Stat is involved in antibacterial immunity, the bacterial clearance and survival rates of shrimp were analyzed in *Stat*-silenced shrimp (Fig. 2G,H). The results showed that the count of *V. anguillarum* increased significantly in *Stat*-silenced shrimp (Fig. 2G). The survival rate of shrimp declined significantly in *Stat*-silenced shrimp compared with *GFP*-silenced shrimp (Fig. 2H). These results suggested that Stat plays an important role in anti-*V. anguillarum* immunity in shrimp.

### Stat phosphorylation increases in βarr1- and TC45-silenced shrimp

**Figure 3:**
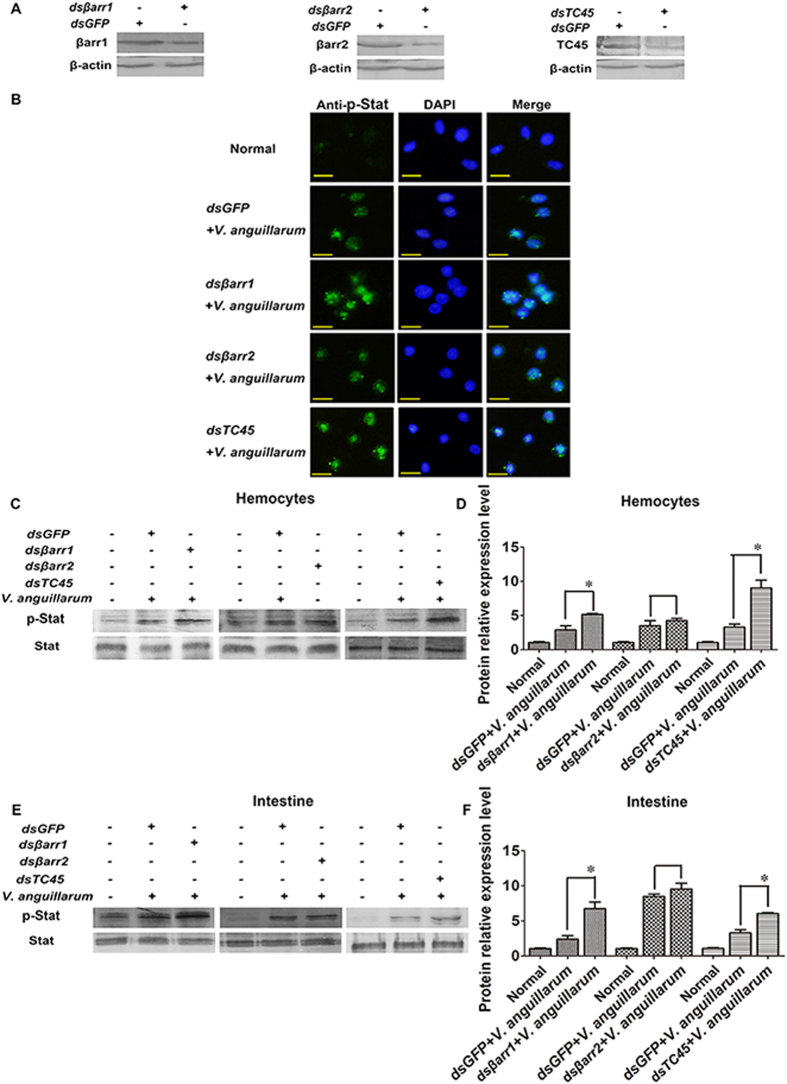
βArr1 and TC45 reduce the phosphorylation of Stat. (**A**) The RNAi efficiency after *dsβarr1*, *dsβarr2* and *dsTC45* injection, as analyzed by western blotting. (**B**) Stat translocation in hemocytes was detected by immunocytochemistry using an antibody recognizing phosphorylated Stat in *βarr1*-, *βarr2-* or *TC45*-silenced shrimp challenged by *V. anguillarum*. (**C**–**F**) Stat phosphorylation in hemocytes (**C**,**D**) and the intestine (**E**,**F**) was detected by western blotting with the antibody recognizing phosphorylated Stat in *βarr1*-, *βarr2-* or *TC45-*silenced shrimp challenged by *V. anguillarum*. (**D**,**F**) separately, are the statistical analysis of (**C**,**E**) after digitization using Quantity One. *dsGFP* injection was used as the control. The experiments were repeats three times.

Stat phosphorylation was analyzed in *βarr1-, βarr2-* or *TC45*-silenced shrimp to study whether βarr1, βarr2 and TC45 affect Stat phosphorylation (Fig. 3A). Immunohistochemistry showed that the level of phosphorylated Stat (p-Stat) in the nucleus increased in hemocytes of *βarr1- and TC45*-silenced shrimp, while there was almost no effect in *βarr2-*silenced shrimp challenged with *V. anguillarum* (Fig. 3B). Western blotting analysis in hemocytes also showed the same results as the immunocytochemistry (Fig. 3C,D). To further confirm the results, we detected the level of p-Stat in the intestines of *βarr1-, βarr2-* or *TC45*-silenced shrimp using western blotting. Same results were obtained as those in hemocytes (Fig. 3E,F). These results suggest that βarr1 and TC45, but not βarr2, inhibit Stat phosphorylation in shrimp.

### βArr1 and TC45 negatively regulate Stat’s antibacterial function by dephosphorylation of Stat

**Figure 4:**
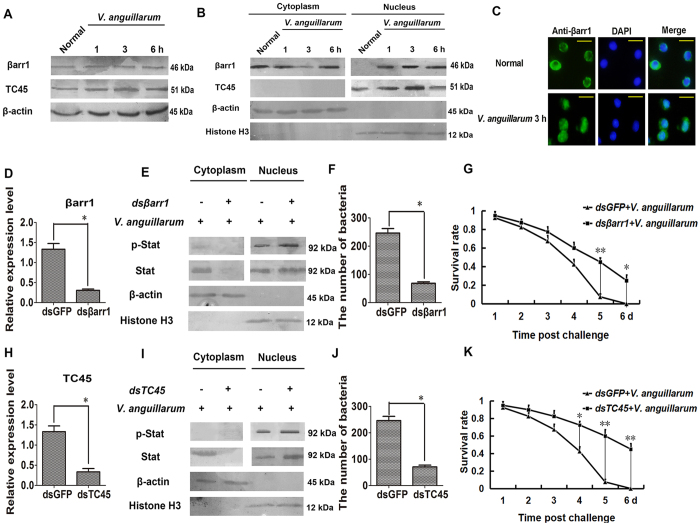
βArr1 and TC45 regulate negatively Stat’s antibacterial function. (**A**) The expressions of βarr1 and TC45 in the hemocytes of shrimp challenged with *V. anguillarum*, as analyzed by western blotting. (**B**) The distribution of βarr1 and TC45 in the nucleus and cytoplasm of hemocytes challenged by *V. anguillarum* at 1, 3, 6 h, as analyzed by western blotting. (**C**) βArr1 translocated into nucleus of hemocytes from shrimp challenged by *V. anguillarum* at 3 h detected by immunocytochemistry using an βarr1 antibody. (**D**,**H**) The RNAi efficiency after *dsβarr1* and *dsTC45* injection, as analyzed by qRT-PCR. (**E**,**I**) Phosphorylated Stat in the hemocyte nucleus and cytoplasm, as detected by western blotting with an anti-Stat antibody in *βarr1*- or *TC45*-silenced shrimp challenged by *V. anguillarum*. *dsGFP* was used as the control. (**F**,**J**) Bacterial clearance was detected in *βarr1-* or *TC45-*silenced shrimp. *dsGFP* was used as the control. (**G**,**K**) The survival rates of *βarr1-* or *TC45-*silenced shrimp challenged by *V. anguillarum*. *dsGFP* was used as the control. The experiments were repeats three times. Differences between groups were analyzed using one-way ANOVA, followed by Tukey’s multiple comparison tests. Significant differences are shown (*p < 0.05, **p < 0.01).

To detect if βarr1 and TC45 are involved in Stat’s anti-*V. anguillarum* response, the protein expression patterns, and subcellular distributions of βarr1 and TC45 were detected by western blotting after challenge with *V. anguillarum* at 1, 3 and 6 h. The results showed no change in the βarr1 expression pattern (Fig. 4A); however, it mainly existed in the cytoplasm in normal shrimp but increased in the nucleus in *V. anguillarum* challenged shrimp at 1, 3 and 6 h (Fig. 4B,C). The expression of TC45 increased at 3 h in *V. anguillarum* challenged shrimp and was present mainly in the nucleus (Fig. 4A,B). We then analyzed the level of p-Stat in hemocytes of *βarr1*-silenced and *TC45*-silenced shrimp (Fig. 4D,H). The results revealed that p-Stat in nucleus increased in *βarr1-* or *TC45*-silenced shrimp challenged by *V. anguillarum* (Fig. 4E,I). Bacterial clearance increased and the number of *V. anguillarum* decreased significantly in *βarr1-* or *TC45*-silenced shrimp (Fig. 4F,J). The survival rates of shrimp increased significantly in *βarr1-* or *TC45*-silenced shrimp compared with *GFP*-silenced shrimp (Fig. 4G,K). These results suggested that βarr1 and TC45 negatively regulate Stat’s antibacterial function by dephosphorylating Stat.

### βArr1, TC45 and Stat interact with each other

**Figure 5:**
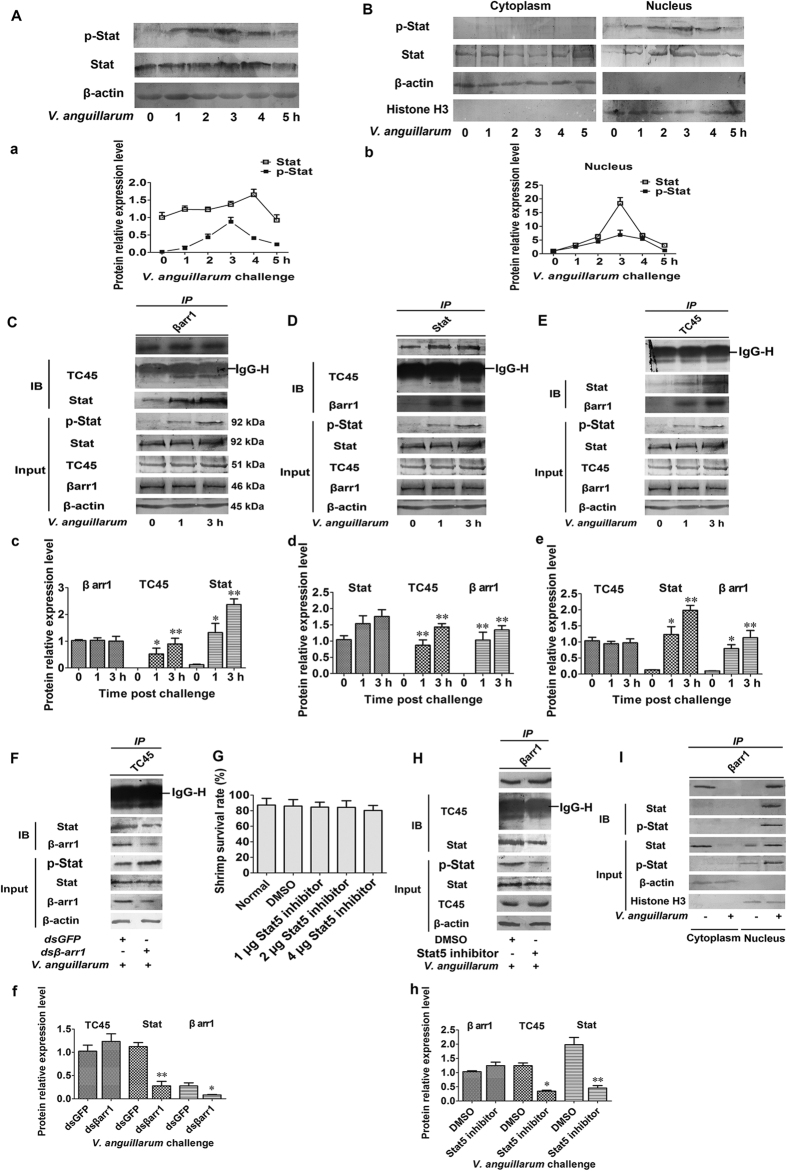
βArr1 interacts with Stat and TC45 to form a heterotrimeric complex. (**A**-a) The total Stat and p-Stat protein level were detected in hemocytes after *V. anguillarum* challenge from 1 to 5 h. (a) is the statistical analysis of A after digitization using Quantity One. (**B**-b) Cytoplasmic and nuclear proteins extracted from hemocytes were used to detect the distribution of Stat and p-Stat in the nucleus and cytoplasm after *V. anguillarum* challenge from 1 to 5 h. (b) is the statistical analysis of B after digitization using Quantity One. **(C**-c) Co-IP was performed using an anti-βarr1 antibody in hemocytes; TC45, Stat, and phosphorylated-Stat (p-Stat) were detected by their corresponding antibodies. (c) is the statistical analysis of C after digitization using Quantity One. (**D**-d) Co-IP was performed using an anti-Stat antibody in hemocytes; TC45, βarr1, and p-Stat were detected by their corresponding antibodies. (d) is the statistical analysis of D after digitization using Quantity One. (**E**-e) Co-IP with an anti-TC45 antibody in hemocytes; βarr1, Stat and p-Stat were detected by their corresponding antibodies. (e) is the statistical analysis of E after digitization using Quantity One. (**F**-f) Co-IP with an anti-TC45 antibody using hemocytes from *βarr1*-silenced shrimp; βarr1, Stat, and p-Stat were detected by their corresponding antibodies. (f) is the statistical analysis of (**F**) after digitization using Quantity One. (**G**) The effect of Stat5 inhibitor on the viability of shrimp. Shrimp were treated with increasing concentrations of Stat5 inhibitor for 2 days and the survival rate was calculated. DMSO was used as the control. (**H**-h) Co-IP with an anti-βarr1 antibody using hemocytes from the Stat5 inhibitor-injected shrimp; TC45, Stat, and p-Stat were detected by their corresponding antibodies. (h) is the statistical analysis of (**H**) after digitization using Quantity One. (**I**) Co-IP with an anti-βarr1 antibody using cytoplasmic and nuclear proteins from hemocytes challenged with *V. anguillarum* at 3 h; Stat and p-Stat were detected by their corresponding antibodies. The experiments were repeats three times. Differences between groups were analyzed using one-way ANOVA, followed by Tukey’s multiple comparison tests. Significant differences are shown (*p < 0.05, **p < 0.01).

The expression pattern of total Stat and p-Stat in hemocytes after *V. anguillarum* challenge from 1 to 5 h was firstly analyzed by western blot. The results showed that total Stat was high expressed in unchallenged shrimp, but relatively increased from 3 to 4 h after bacterial challenge, and almost no p-Stat was detected in unchallenged shrimp, but the p-Stat was increased from 1 to 3 h and then recovered at 5 h after bacterial challenge (Fig. 5A-a). Then cytoplasmic and nuclear proteins from hemocytes were extracted respectively for western blot analysis, and the results showed that total Stat was existed in normal shrimp and decreased from 1 to 4 h and recovered at 5 h after bacterial challenge in cytoplasm. However, nearly no p-Stat was detected in cytoplasm of the hemocytes from normal and bacterial challenged shrimp (Fig. 5B). Conversely, the expression of total Stat was increased from 1 to 4 h after *V. anguillarum* challenge in nucleus, and p-Stat was also increased from 1 to 3 h and then decreased from 4 to 5 h post bacterial challenge in nucleus (Fig. 5B-b). These results suggested that bacterial challenge induced Stat phosphorylation and translocation into nucleus leading to a clear increase of p-Stat in nucleus at early stage of bacterial challenge, and the p-Stat was dephosphorylated leading to obvious decrease at late stage of bacterial challenge. To analyze possible machanism, the Co-IP assays were performed to study the interactions of βarr1 and TC45 with Stat in hemocytes using antibodies against βarr1, TC45 or Stat. The results showed that βarr1, TC45 and Stat interacted with each other and the interaction was enhanced following the increase of Stat phosphorylation (Fig. 5C–E). The interaction of TC45 with Stat became weak in *βarr1*-silenced shrimp after challenge with *V. anguillarum* (Fig. 5F-f). To further confirm above results, Stat5 inhibitor was used to inhibit Stat phosphorylation. Initially, the toxic effect of Stat5 inhibitor on shrimp was determined by analyzing the survival rate of shrimp after Stat5 inhibitor injection. The results showed that low doses of Stat5 inhibitor ( < 4 μg per shrimp) did not reduce the viability of shrimp (Fig. 5G). After Stat5 inhibitor injection, Co-IP with βarr1 antibody was performed using hemocytes. The results showed that the interaction of βarr1, TC45 and Stat became weak after injection of Stat5 inhibitor (Fig. 5H-h). As p-Stat could be translocated into the nucleus, the nuclear and cytoplasmic proteins were extracted and the Co-IP assays were performed. The result showed βarr1 could interact with Stat in the nucleus but not in the cytoplasm (Fig. 5I). The results further confirmed that the nuclear βarr1 directly interacts with Stat. The above results suggested that βarr1 acts as a scaffold protein to recruit the tyrosine phosphatase TC45 onto phosphorylated Stat to dephosphorylate Stat in the nucleus.

### The C-terminal domain of βarr1 interacts with the Link domain of Stat

**Figure 6:**
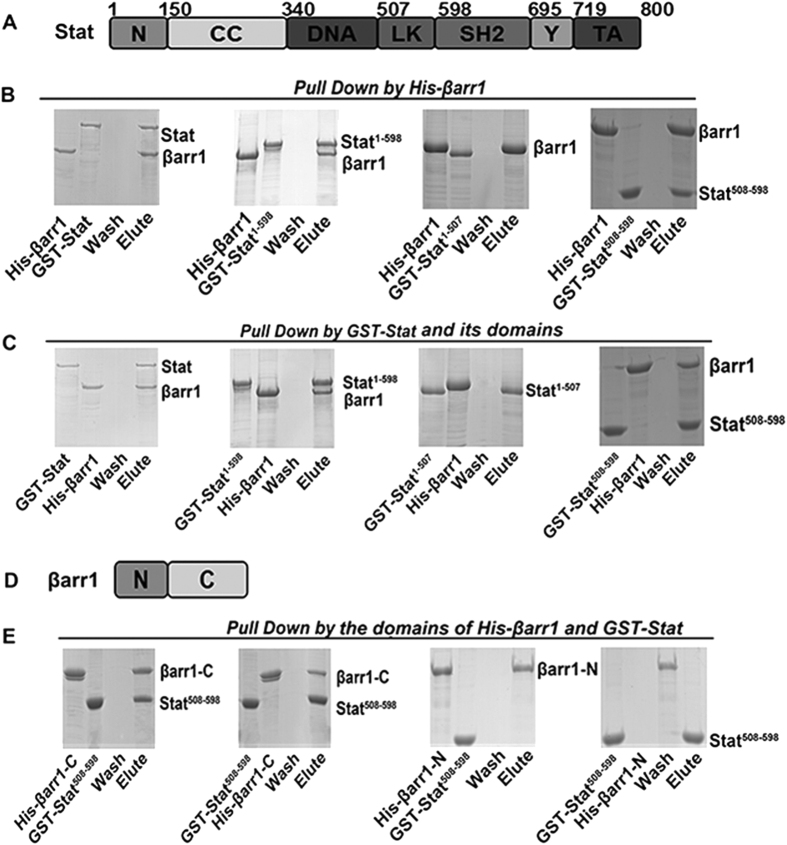
βArr1 interacts with Stat. (**A**) Domain architectures of Stat. (**B**) His-pull-down assays were performed to detect the interaction of His-βarr1 with different domains of GST-Stat. (**C**) GST-pull-down assays were performed to detect the interaction of different domains of GST-Stat with His-βarr1. (**D**) Domain architectures of βarr1. (**E**) His- and GST-pull-down assays were performed to detect interaction of His-βarr1-C with Stat^508–598^ of GST-Stat. The experiments were repeats three times.

*M*. *japonicus* Stat contains N-terminal domain (NTD; aa 1–150), a coiled-coil domain (CC; aa 151–340), a DNA-binding domain (DB; aa 341–507), a linker domain (LK; aa 508–598), an SH2 domain (SH2; aa 599–695), a tyrosine phosphorylation site (Y site; aa 696–719) and a transactivation domain, TAD, (aa 720–800) (Fig. 6A). To detect which domain of Stat is responsible for the interaction with βarr1, recombinant proteins of Stat and its individual domains with GST-tags (Stat, 1–598, 1–507, 508–598) (Fig. 6A), and βarr1 and its different domains with His-tags βarr1, βarr1–N (N-terminal domain), βarr1–C (C-terminal domain) (Fig. 6D) were used in pull-down assays. As shown in Fig. 6B,C, the LK domain of Stat interacted with βarrl. Recombinant βarrl, βarr1–N and βarr1–C were used for pull-down assays to analyze the interaction with LK domain of Stat (Fig. 6E). The results showed that LK domain of Stat interacts with the C-terminal domain of βarr1.

### TC45 interacts with Stat^599–800^ and the C-terminal domain of βarr1

**Figure 7:**
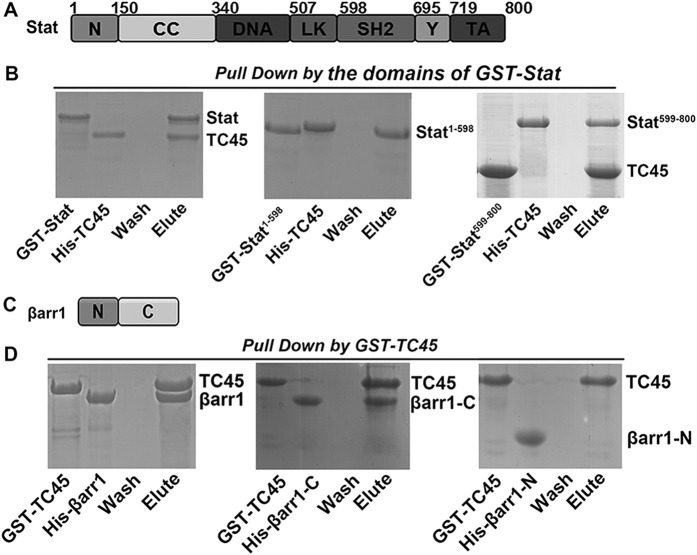
TC45 interacts with Stat and βarr1. (**A**) Domain architecture of Stat. (**B**) GST-pull-down assays were performed to detect the interaction of GST-TC45 with different domains of His-Stat. (**C**) Domain architecture of βarr1. (**D**) The interaction of GST-TC45 with different domains of His-βarr1 was analyzed by a pull-down assay. The experiments were repeats three times.

To study which domain of Stat interacts with TC45, His-tagged TC45 was also expressed in *E. coli*. The results showed TC45 could interact with Stat^599–800^ which contains the Tyr phosphorylation sites (Fig. 7B). We expressed TC45 with a GST-tag, and GST-pull-down with βarr1 was performed. The results showed βarr1 interacted with TC45 via its C-terminal domain (Fig. 7D). Therefore, βarr1 could interact with the LK domain of Stat and with TC45 via its C-terminal domain.

### βArr1 and TC45 affect the expressions of AMPs that are regulated by Stat

**Figure 8:**
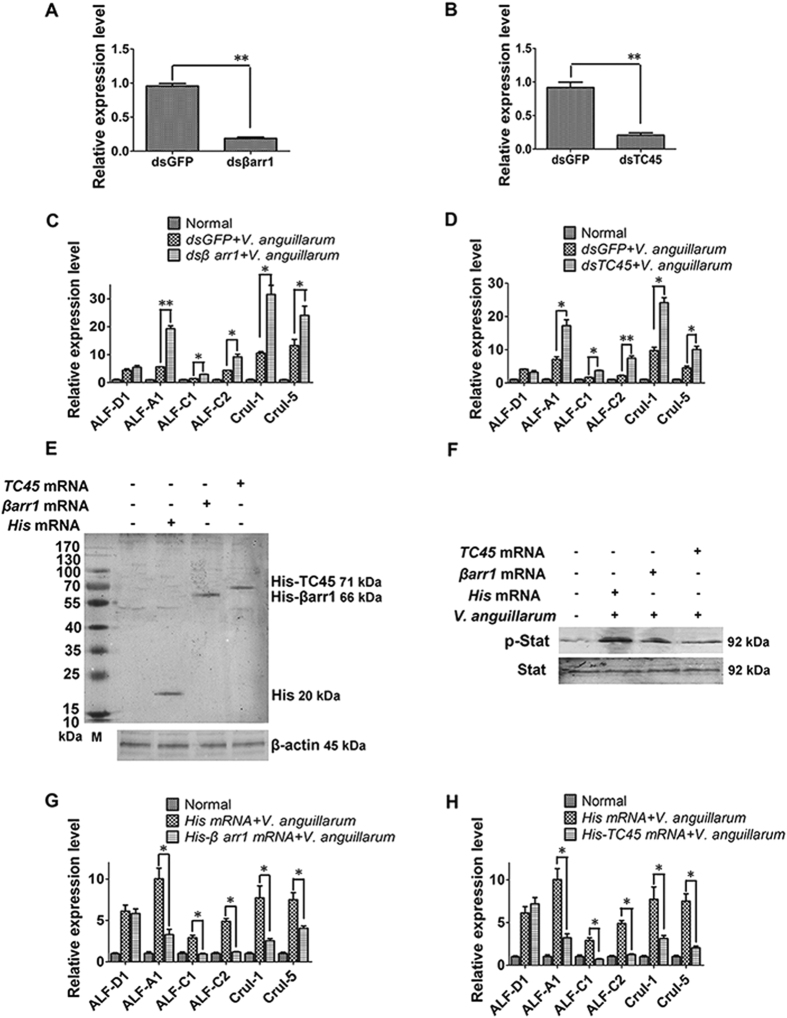
βArr1 and TC45 are involved in the regulation of AMP expression. (**A**,**B**) RNAi efficiency after *dsβarr1* and *dsTC45* injection, as detected by qRT-PCR. (**C**,**D**) qRT-PCR to detect the expression of *CruI-1*, *CruI-5*, *ALF-A1*, *ALF-C1*, *ALF-C2* and *ALF-D1* in *βarr1* or *TC45*-silenced shrimp challenged by *V. anguillarum* at 6 h. (**E**) Western blotting to analysis of βarr1 or TC45 expression in hemocytes from mRNA-injected shrimp. Hemocytes were extracted for western blotting 24 h post mRNA injection. An anti-His-tag monoclonal antibody was used as the primary antibody. (**F**) Stat phosphorylation was detected in *βarr1-* and *TC45*-overexpressing shrimp after challenge with *V. anguillarum* at 3 h. (**G**,**H**) qRT-PCR was used to detect the expressions of *CruI-1*, *CruI-5*, *ALF-A1*, *ALF-C1*, *ALF-C2* and *ALF-D1* in *βarr1-* and *TC45*-overexpressing shrimp challenged by *V. anguillarum* at 6 h. The experiments were repeats three times. Differences were analyzed using one-way ANOVA, followed by Tukey’s multiple comparison tests. The significant differences are shown (*p < 0.05, **p < 0.01, ***p < 0.001).

To study if the AMPs regulated by Stat are also affected by βarr1 and TC45, the expression of five AMPs was detected in *βarr1-* or *TC45*-silenced shrimp challenged with *V. anguillarum* (Fig. 8A,B). The results showed that the expression of *CruΙ-1*, *CruΙ-5*, *ALF-A1*, *ALF-C1*, and *ALF-C2* increased significantly in *βarr1-* or *TC45*-silenced shrimp after challenge with *V. anguillarum* at 6 h (Fig. 8C,D).

To further confirm the results, *βarr1-* or *TC45*-overexpression assays were conducted. The results showed that Stat phosphorylation was inhibited in *βarr1-* or *TC45*-overexpressing shrimp challenged with *V. anguillarum* (Fig. 8E,F). The expression of *CruΙ-1*, *CruΙ-5*, *ALF-A1*, *ALF-C1*, and *ALF-C2* declined significantly in *βarr1-* and *TC45*-overexpressing shrimp after challenge with *V. anguillarum* at 6 h (Fig. 8G,H). These results suggested that βarr1 and TC45 negatively regulate AMP expression.

## Discussion

Stats, as important transcription factors, are regulated tightly in animals. In *Drosophila*, Stat92E is activated by Upds, Dome, Jak and inhibited by Socs36E^29^, ken^30^, PIAS^31^, and NURF^32^. SOCSs regulate Stat activation by inhibiting Jak^29^. Ken and NURF inhibit Stat activation in the nucleus by directly blocking the binding of Stat to its targets^30^. PIAS can interact directly with p-Stat to inhibit Stat activation^31^. In shrimp, a Dome^42^, a Jak^43^ and a Stat^38^,^39^ in the Jak/Stat pathway have been identified. Shrimp Stat is similar to mammalian Stat5a/b. In mammals, Jak2 and 3^44^,^45^ regulate Stat5a/b and the inhibitors are Socs2^46^, TC-PTP^47^ and PTP1B^48^. The inhibitor SOCS2 has also been cloned in shrimp^38^. In the present study, we found that Stat was activated by *V. anguillarum* challenge and the activated Stat was negatively regulated by βarr1 and TC45 in shrimp.

βArrs are multifunctional signaling molecules that can affect multiple signal pathways, such as GPCR signal transduction^33^, and the activities or subcellular distribution of signaling molecules^34^. Recent studies suggested that βarr1 in the nucleus binds and recruits histone acetylase p300 to specific genome promoters and regulates gene transcription in mammals^49^. Moreover, in mammals, βarr1 also recruits tyrosine phosphatase TC45 to Stat1 in the nucleus in response to IFN-gamma stimulation and affects Stat1 phosphorylation^35^. In shrimp, two β-arrestins (βarr 1 and 2) were identified that regulated the Toll pathway negatively by inhibiting Dorsal translocating into the nucleus^40^. In the present study, βarr1, but not βarr2, was observed to regulate specifically tyrosine phosphorylation of Stat in shrimp, resulting in decreased AMP expression. βArr1 is a scaffold protein; therefore, how does it function to regulate tyrosine phosphorylation of Stat ? 

TC45 is the most important tyrosine phosphatase in the process of Stat1 dephosphorylation in mammals^23^. PTPs regulate Stat3 activity negatively, either in the cytoplasm, through dephosphorylating protein tyrosine kinase Jak (for instance, PTPTC48)^21^, or in the nucleus, through directly dephosphorylating Stat3 (for instance, TC45)^14^. Moreover, TC45 is not a Stat3-specific phosphatase; it also mediates the dephosphorylation of other Stat family, members such as Stat1^22^,^23^. GdX, as a TC-PTP cofactor, bridges the association of TC45 with Stat3 specifically to mediate Stat3 dephosphorylation, which blocks Stat3-P-dependent cancer cell growth in mammals^37^. However, whether tyrosine phosphatase TC45 could affect Stat dephosphorylation in invertebrate nucleus is still unknown. In this paper, we found that in the nucleus, TC45 could interact with Stat in nucleus to dephosphorylate Stat, resulting in decreased AMP expression.

**Figure 9:**
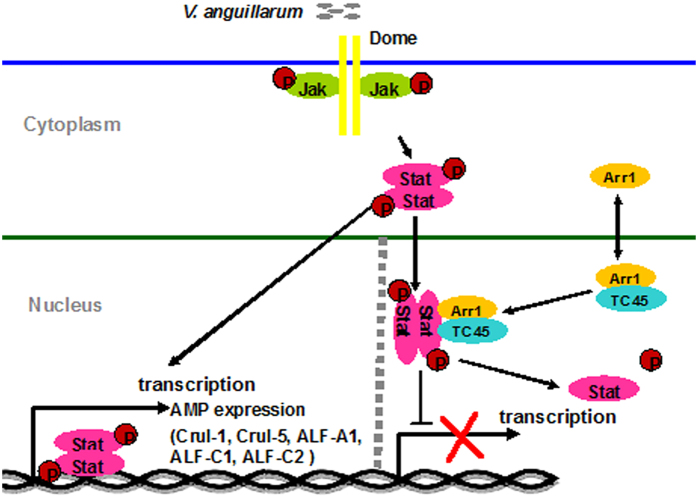
A model of Stat signaling attenuation in the nucleus. Stat phosphorylation and transcription are activated by *V. anguillarum*, followed by induction of AMP (CruI-1, CruI-5, ALF-A1, ALF-C1, ALF-C2) expression. βArr1 interacts with TC45 to dephosphorylate Stat in the nucleus. Thus, the TC45/βarr1 complex promotes Stat dephosphorylation to inhibit Stat transcription activity, thereby attenuating AMP expression in the nucleus.

In summary, *V. anguillarum* infection activates Stat signaling by inducing tyrosine phosphorylation of Stat. p-Stat is then translocated into the nucleus to regulate AMP expression. The scaffold protein βarr1 bridges TC45 and Stat in nucleus, allowing TC45 to dephosphorylate Stat, thereby inhibiting Stat’s transcription activity (Fig. 9). Our results demonstrated that in shrimp, TC45 and βarr1 are negative regulators in Stat signaling.

## Materials and Methods

### Tissue distribution

Kuruma shrimp *Marsupenaeus japonicus* (8–10 g each) from a fishery market in Jinan, Shandong Province, China were cultured in laboratory aquarium tanks with aerated seawater at 22 °C. After acclimatization for 2 days, five organs from at least three normal shrimp, including the heart, hepatopancreas, gills, stomach and intestine, were collected. Total RNA was extracted using the Trizol reagent (Cwbio, Beijing, China) and proteins were obtained using a lysis buffer (150 mM NaCl, 1.0% Nonident-P40, 0.1% SDS, 50 mM Tris [pH 8.0] containing a protease inhibitor cocktail (Abcam USA). For hemocytes collection, the hemolymph was extracted from at least three shrimp using a syringe containing 1 ml of anticoagulant buffer (0.45 M NaCl, 10 mM KCl, 10 mM EDTA and 10 mM HEPES, pH 7.45) and immediately centrifuged at 800 × g for 10 min (4 °C).

The mRNA distribution of four genes, *βarr1*, *βarr2*, *TC45* and *Stat*, in hemocytes, heart, hepatopancreas, gills, stomach and intestine was analyzed using quantitative real-time reverse transcription polymerase chain reaction (RT-PCR) with the primers RT-F and RT-R for the above four genes (Table 1). *β-actin* was used as the control with the primers β-actinF and β-actinR (Table 1). All experiments were repeated at least three times using individual templates. The qRT-PCR program was: 95 °C for 5 min; followed by 40 cycles at 95 °C for 10 s, 60 °C for 30 s, and 72 °C for 20 s. The plate was read at 78 °C for each cycle. The final product was analyzed via a DNA melting analysis from 65 °C to 95 °C. The obtained data were analyzed statistically and their relative expressions were calculated using the 2^−ΔΔCt^ method, as described previously^50^. Differences in the unpaired sample *t* test were considered significant at *P < 0.05, **P < 0.01, ***P < 0.001. The proteins of βarr1, βarr2, TC45 and Stat from hemocytes and five organs were analyzed by western blotting with their corresponding antibodies.

### Molecular cloning and sequence analysis

The full-length cDNA sequences of *TC45* and *Stat* were obtained from transcriptomic sequencing of *M. japonicus*. The sequences of *βarr1* and *βarr2* were reported in our previous paper^40^. Similarity analysis was conducted using BLASTx (http://www.ncbi.nlm.nih.gov/). The cDNA sequences *TC45* and *Stat* were conceptually translated and the corresponding deduced proteins were predicted using ExPASy (http://www.expasy.org/). The domain architecture prediction of the proteins was analyzed using SMART (http://smart.embl-heidelberg.de/).

### Recombinant expression and antiserum preparation

*M*. *japonicus* Stat contains an NTD, CC, DB, LK, SH2, Y site and TAD. DNAs encoding the full length Stat, and fragments 1–507, 1–598, 508–598 and 599–800 of Stat were recombinantly and separately expressed in *Escherichia coli* as GST-tagged proteins. βarr1, βarr1-N, βarr1-C and βarr2 were recombinantly expressed with His-tags and GST-tags; TC45 was recombinantly expressed as a GST-tagged protein, all in *E. coli*. The sequences encoding the above proteins were amplified using the primers ExF and ExR (Table 1). The PCR products were inserted into vector pET32a (Novagen) or pGEX4T-1 (GE Healthcare). The recombinant proteins were purified by affinity chromatography using His-Bind resin (Ni2 + -resin; Novagen, Darmstadt, Germany) or GST-resin (GenScript, Nanjing, China), following the manufacturers’ instructions. Antiserum preparation was performed as previously described^51^.

### RNA interference

The cDNA fragments of *βarr1*, *βarr2*, *TC45* and *Stat* amplified by primers Fi and Ri (Table 1) were used as templates for the synthesis of dsRNA (double strand RNA). The cDNA fragment of *GFP* used for *dsGFP* synthesis was amplified using primers GFP-Fi and GFP-Ri (Table 1). The dsRNA was synthesized using T7 polymerase (Fermentas, USA) based on a previous method^52^. dsRNA (20 μg) of *βarr1*, *βarr2*, *TC45* or *Stat* was injected into the abdominal segment of shrimp. To enhance the RNAi effect, a second injection was performed 12 h after the first injection. The *dsGFP* was used as the control. The hemocytes were collected from the shrimp 24 h after the second injection, and total RNA was extracted for RNAi efficiency analysis by RT-PCR using primers RT-F and RT-R (Table 1). Total protein was also extracted. The experiments were repeated three times.

### Bacterial clearance and Survival rate assay

The bacterial clearance assay was performed in *βarr1*-, *TC45*- and *Stat*-silenced shrimp. Shrimp were separated into five groups, and injected with *dsβarr1*, *dsTC45* and *dsStat*, or injected with *dsGFP* as a control. The four groups were then injected with *V. anguillarum* (20 μl, 1 × 10^8^ CFU). One hour after bacteria injection, shrimp hemolymph was collected and diluted in PBS (140 mM NaCl, 10 mM sodium phosphate, pH 7.4) (1/10000), and then cultured on LB solid plates overnight in 37 °C. The number of bacterial colonies was counted. The assay was repeated three times.

For the survival assay, shrimp (8–10 g each individual, 30 shrimp per group) were divided into four groups, and injected with *dsβarr1*, *dsTC45* or *daStat*; The *dsGFP* injection group was used as the control. The gene-silenced shrimp and the control group were infected with *V. anguillarum* (20 μl, 1 × 10^8^ CFU) and shrimp mortality was detected. The number of dead shrimp was monitored every day. The survival rate of the each group was calculated. The experiments were repeated three times.

### Western blotting analysis

Proteins were extracted from the hemocytes and intestine of normal shrimp and bacterial challenged shrimp. Cytoplasmic and nuclear proteins were extracted, the latter using a Nuclear Protein Extraction Reagent Kit (BioTeke, China), following the manufacturer’s instruction. The obtained samples were separated by 10% SDS-polyacrylamide gel electrophoresis. The proteins in the gel were then transferred onto a nitrocellulose membrane. The membrane was then blocked for 2 h with 3% non-fat milk in TBST (10 mM Tris-HCl, pH 7.5, 150 mM NaCl, 0.2% Tween-20), and incubated with 1/100 diluted antiserum against βarr1, βarr2, TC45, Stat, p-Stat or β-actin in TBST with 3% non-fat milk for 3 h. Alkaline phosphatase-conjugated goat anti-rabbit IgG (1/10,000 diluted in TBS) was added after washing out nonspecific binding antiserum with TBST and incubation continued for 2 h. The membrane was dipped in the reaction system (10 ml of TBS, with 45 μl of NBT and 35 μl of BCIP) in the dark for 5–20 min at 37 °C to visualize the signal.

### Immunocytochemistry

Hemolymph from normal or challenged shrimp was fixed with a 1 ml mixture of anticoagulant (pH 7.4) and 4% paraformaldehyde (1:1) and then centrifuged at 600 × g for 3 min at 4 °C. Collected hemocytes were washed three times with PBS and then deposited on a glass slide, washed with PBS and then incubated in 0.2% triton at 37 °C for 5 min. After washing with PBS, the hemocytes on the glass slide were blocked with 3% BSA (30 min, 37 °C), and incubated with an antibody recognizing phosphorylated Stat (anti-p-Stat) (1:400 in 3% BSA) overnight at 4 °C. On the second day, the glass slide with the hemocytes was washed with PBS, and incubated with 3% BSA for 10 min, and then with Alexa fluor 488-conjugated anti-rabbit secondary antibody (1:800 ratio, diluted in 3% BSA) for 1 h at 37 °C in the dark. After washing six times, the hemocytes were incubated with 4′-6-diamidino-2-phenylindole dihydrochloride (DAPI, AnaSpec Inc., San Jose, CA, USA; 1 μg/ml in PBS) for 10 min at room temperature and then washed six times. Hemocytes on the slide were observed under an Olympus BX51 fluorescence microscope (Shinjuku-ku, Tokyo, Japan).

### Co-immunoprecipitation (Co-IP) assay

Proteins from hemocytes were collected with lysis buffer containing a protease inhibitor cocktail (Merck, Gemery) and incubated with protein A for 10 min to remove non-specific binding proteins. The proteins were then incubated with the antibodies of interest over night at 4 °C. On the second day, the mixture (protein and antibody) was incubated with protein A for 3 h at room temperature and then washed with PBS four times. The obtained mixture (bound protein, antibody and protein A) was analyzed by western blotting.

### Inhibitor injection

Stat from shrimp is similar to human Stat5; therefore, Stat5 inhibitor from Merck was used to inhibit shrimp Stat. To ascertain the effect of Stat5 inhibitor on shrimp viability, the survival rate of shrimp after inhibitor injection was analyzed. Shrimp (8–10 g each individual, 20 shrimp per group) were divided into four groups, and injected with 1, 2 and 4 μg of Stat5 inhibitor, respectively. The dimethylsulfoxide (DMSO) injection group was used as the control. For the Co-IP assay, Stat5 inhibitor (2 μg) was injected into each shrimp, which were then challenged with *V. anguillarum*. DMSO injection was used as a control. The hemocyte proteins were collected for protein extraction 3 h after *V. anguillarum* challenge to further detect if Stat inactivation affected the interaction of βarr1 with Stat and TC45.

### Pull-down assays

The recombinant proteins tagged with a GST-tag (30 μg) were added into 20 μl of Glutathione resin and incubated for 2 hours at room temperature with slight rotation. The mixture (resin and binding protein) was washed with TBS three times by centrifugation at 500 × g for 3 min to remove the unbound proteins. The His-tagged test protein was added into the mixture containing the Glutathione resin, and gently rotated at room temperature for 2 h. After washing three times, the mixture was analyzed by SDS-PAGE. The pull-down assay for His-tag proteins was performed the same as that for GST-tagged proteins.

### AMP expression in Stat-, βarr1- and TC45-silenced shrimp challenged with *V. anguillarum*

Shrimp were divided into four groups and injected with *dsGFP*, *dsStat*, *dsβarr1* and *dsTC45*, separately. AMPs including *CruΙ-1*, *CruΙ-5*, *ALF-A1*, *ALF-C1*, *ALF-C2* and *ALF-D1*^53^ were detected by qRT-PCR with corresponding primers (Table 1) after 6 h of *V. anguillarum* challenge. Shrimp injected with *dsGFP* were used as the control.

### Overexpression of βarr1 and TC45

The pET32a vectors containing the *βarr1* and *TC45* genes were digested with restriction enzyme *Xho*I. The cleaved vectors were used as template to transcribe the capped *βarr1* and *TC45* mRNA using ta T7 RNA polymerase *in vitro* transcription kit (Ambion, Inc.) according to the manufacturer’s instructions. The empty pET32a vectors were also digested by *Xho*I and used as a template to transcribe Trx-tag and His-tag sequences for use as controls. The mRNA for *βarr1* and *TC45* (at a concentration of 6 μM) with His-tag sequences and the control sequences were injected separately into shrimp. After 24 h of mRNA injection, the hemocyte proteins of each group were extracted for western blotting analysis using an anti-His-tag monoclonal antibody to detect the efficiency of overexpression. The proteins extracted from hemocytes were also used for western blotting to detect Stat phosphorylation in *βarr1*- or *TC45*-overexpressing shrimp after challenge with *V. anguillarum* for 3 h. The *V. anguillarum* were injected into shrimp 6 h post mRNA injection, and total RNA was isolated from shrimp hemocytes using the Trizol reagent and used for reverse transcription of cDNA to detect AMP expression.

## Additional Information

**How to cite this article**: Sun, J.-J. *et al*. β-Arrestin 1’s Interaction with TC45 Attenuates Stat signaling by dephosphorylating Stat to inhibit antimicrobial peptide expression. *Sci. Rep.* **6**, 35808; doi: 10.1038/srep35808 (2016).

**Publisher’s note:** Springer Nature remains neutral with regard to jurisdictional claims in published maps and institutional affiliations.

## Figures and Tables

**Table 1:** Sequences of the primers used in this study.

Primer	Sequence (5′–3′)
Tissue distribution and expression pattern analysis	
βarr1-RT-F	TTTCACGCTGACGCCACT
βarr1-RT-R	AGCAACCAGATCACCAACTAG
βarr2-RT-F	TGGCTCTATTCCTCTGCG
βarr2-RT-R	TGGCTCTATTCCTCTGCG
TC45-RT-F	CTCCCTTACCCGCTCTATGA
TC45-RT-R	TTTCTCTTGCCAATGTTCGT
Stat-RT-F	GGTCCCAGTTCTGTAAGG
Stat-RT-R	TAGGCACATTCGGATAAA
β-actin F	CAGCCTTCCTTCCTGGGTATGG
β-actin R	GAGGGAGCGAGGGCAGTGATT
ALF-A1-RT-F	CTGGTCGGTTTCCTGGTGGC
ALF-A1-RT-R	CCAACCTGGGCACCACATACTG
ALF-C1-RT-F	CGCTTCAAGGGTCGGATGTG
ALF-C1-RT-R	CGAGCCTCTTCCTCCGTGATG
ALF-C2-RT-F	TCCTGGTGGTGGCAGTGGCT
ALF-C2-RT-R	TGCGGGTCTCGGCTTCTCCT
ALF-D1-RT-F	GCTTTTTATTTTGGGGGTCACGCTGT
ALF-D1-RT-R	CTTTGGCGTGGAACAAGGTAGAGGAT
CruI-1-RT-F	TGCTCAGAACTCCCTCCACC
CruI-1-RT-R	TTGAATCAGCCCATCGTCG
CruΙ-5-RT-F	TGCGAAACAGACAGGGATTGC
CruΙ-5-RT-R	CCGAAGACCAGATGACCGAAA
Recombinant expression	
βarr1-ExF	TACTCAGGATCCATAATGGAGGACAACAG
βarr1-ExR	TACTCACTCGAGTTCCTACGCCTCTGT
βarr1-C-ExF	TACTCAGGATCCATAATGGAGGACAACAGC
βarr1-C-ExR	TACTCACTCGAGGCATACATGACCTTCCTG
βarr1-N-ExF	TACTCAGGATCCCAAGGAGAACAGCCAAGTGTG
βarr1-N-ExR	TACTCACTCGAGTTCCTACGCCTCTGTTTCTCC
TC45-ExF	TACTCAGAATTCGTCAGTATGCCTACAAATCTA
TC45-ExR	TACTCACTCGAGCTGGACACGAGGACGCTT
Stat(1aa)-ExF	TACTCAGAATTCATGTCGTTGTGGAACAGAGC
Stat(800aa)-ExR	TACTCACTCGAGTTACAAATGCCGGTGAACATA
Stat(507aa)-ExR	TACTCACTCGAGAGCATTATCCCATGA
Stat(507aa)-ExF	TACTCAGGATCCACAGTGTCATGGGAT
Stat(598aa)-ExR	TACTCACTCGAGCAGCATCTCTTCAGC
Stat(598aa)-ExF	TACTCACTCGAGAAGTACTACACGCCA
RNA interference	
βarr1-Fi	GCGTAATACGACTCACTATAGGCTGATTCAAGTCAAAAGGAG
βarr1-Ri	GCGTAATACGACTCACTATAGGGAATCTTCCTTTCTGTTCAAC
βarr2-Fi	GCGTAATACGACTCACTATAGGTCCTCTGCGTGAACATTTTCC
βarr2-Ri	GCGTAATACGACTCACTATAGGCTTCTCTTATATCTCCTCGA
TC45-Fi	GCGTAATACGACTCACTATAGGATGGTGCACATAAGCTA
TC45-Ri	GCGTAATACGACTCACTATAGGCACAATTCATGTAGGA
Stat-Fi	GCGTAATACGACTCACTATAGGGACTTTCCTGCTCCGTTTC
Stat-Ri	GCGTAATACGACTCACTATAGGGCGTTGGCACTGTTGAGAC
GFP-Fi	GCGTAATACGACTCACTATAGGTGGTCCCAATTCTCGTGGAAC
GFP-Ri	GCGTAATACGACTCACTATAGGCTTGAAGTTGACCTTGATGCC

## References

[b1] Darnell J. E. Jr., Kerr I. M. & Stark G. R. Jak-STAT pathways and transcriptional activation in response to IFNs and other extracellular signaling proteins. Science 264, 1415–1421 (1994). 8197455 10.1126/science.8197455

[b2] Ihle J. N. Cytokine receptor signalling. Nature 377, 591–594 (1995). 7566171 10.1038/377591a0

[b3] O’Shea J. J., Gadina M. & Schreiber R. D. Cytokine signaling in 2002: new surprises in the Jak/Stat pathway. Cell 109 Suppl, S121–S131 (2002). 11983158 10.1016/s0092-8674(02)00701-8

[b4] Schindler C. & Plumlee C. Inteferons pen the JAK-STAT pathway. Semin Cell Dev Biol 19, 311–318 (2008). 18765289 10.1016/j.semcdb.2008.08.010 PMC2741134

[b5] Ivashkiv L. B. & Hu X. Signaling by STATs. Arthritis Res Ther 6, 159–168 (2004). 15225360 10.1186/ar1197 PMC464899

[b6] Prenzel N., Fischer O. M., Streit S., Hart S. & Ullrich A. The epidermal growth factor receptor family as a central element for cellular signal transduction and diversification. Endocr Relat Cancer 8, 11–31 (2001). 11350724 10.1677/erc.0.0080011

[b7] Concha-Benavente F., Srivastava R. M., Ferrone S. & Ferris R. L. EGFR-mediated tumor immunoescape: The imbalance between phosphorylated STAT1 and phosphorylated STAT3. Oncoimmunology 2, e27215 (2013). 24501692 10.4161/onci.27215 PMC3913673

[b8] Li W. X. Canonical and non-canonical JAK-STAT signaling. Trends Cell Biol 18, 545–551 (2008). 18848449 10.1016/j.tcb.2008.08.008 PMC3082280

[b9] Hart J. R., Liao L., Yates J. R. 3rd & Vogt P. K. Essential role of Stat3 in PI3K-induced oncogenic transformation. Proc Natl Acad Sci USA 108, 13247–13252 (2011). 21788516 10.1073/pnas.1110486108 PMC3156164

[b10] Garcia R. . Constitutive activation of Stat3 by the Src and JAK tyrosine kinases participates in growth regulation of human breast carcinoma cells. Oncogene 20, 2499–2513 (2001). 11420660 10.1038/sj.onc.1204349

[b11] Yoshimura A., Nishinakamura H., Matsumura Y. & Hanada T. Negative regulation of cytokine signaling and immune responses by SOCS proteins. Arthritis Res Ther 7, 100–110 (2005). 15899058 10.1186/ar1741 PMC1174965

[b12] Yoshikawa H. . SOCS-1, a negative regulator of the JAK/STAT pathway, is silenced by methylation in human hepatocellular carcinoma and shows growth-suppression activity. Nat Genet 28, 29–35 (2001). 11326271 10.1038/ng0501-29

[b13] Ruela-de-Sousa R. R., Queiroz K. C., Peppelenbosch M. P. & Fuhler G. M. Reversible phosphorylation in haematological malignancies: potential role for protein tyrosine phosphatases in treatment ? Biochim Biophys Acta 1806, 287–303 (2010). 20659529 10.1016/j.bbcan.2010.07.007

[b14] Yamamoto T. . The nuclear isoform of protein-tyrosine phosphatase TC-PTP regulates interleukin-6-mediated signaling pathway through STAT3 dephosphorylation. Biochem Biophys Res Commun 297, 811–817 (2002). 12359225 10.1016/s0006-291x(02)02291-x

[b15] Zhang X. . Identification of STAT3 as a substrate of receptor protein tyrosine phosphatase T. Proc Natl Acad Sci USA 104, 4060–4064 (2007). 17360477 10.1073/pnas.0611665104 PMC1802729

[b16] Bard-Chapeau E. A. . Ptpn11/Shp2 acts as a tumor suppressor in hepatocellular carcinogenesis. Cancer Cell 19, 629–639 (2011). 21575863 10.1016/j.ccr.2011.03.023 PMC3098128

[b17] Su F. . Protein tyrosine phosphatase Meg2 dephosphorylates signal transducer and activator of transcription 3 and suppresses tumor growth in breast cancer. Breast Cancer Res 14, R38 (2012). 22394684 10.1186/bcr3134 PMC3446372

[b18] Long J. . Repression of Smad transcriptional activity by PIASy, an inhibitor of activated STAT. Proc Natl Acad Sci USA 100, 9791–9796 (2003). 12904571 10.1073/pnas.1733973100 PMC187844

[b19] Zhang Q. . Multilevel dysregulation of STAT3 activation in anaplastic lymphoma kinase-positive T/null-cell lymphoma. J Immunol 168, 466–474 (2002). 11751994 10.4049/jimmunol.168.1.466

[b20] Ren F. . SIPAR negatively regulates STAT3 signaling and inhibits progression of melanoma. Cell Signal 25, 2272–2280 (2013). 23917203 10.1016/j.cellsig.2013.07.023

[b21] Simoncic P. D., Lee-Loy A., Barber D. L., Tremblay M. L. & McGlade C. J. The T cell protein tyrosine phosphatase is a negative regulator of janus family kinases 1 and 3. Curr Biol 12, 446–453 (2002). 11909529 10.1016/s0960-9822(02)00697-8

[b22] Aoki N. & Matsuda T. A nuclear protein tyrosine phosphatase TC-PTP is a potential negative regulator of the PRL-mediated signaling pathway: dephosphorylation and deactivation of signal transducer and activator of transcription 5a and 5b by TC-PTP in nucleus. Mol Endocrinol 16, 58–69 (2002). 11773439 10.1210/mend.16.1.0761

[b23] ten Hoeve J. . Identification of a nuclear Stat1 protein tyrosine phosphatase. Mol Cell Biol 22, 5662–5668 (2002). 12138178 10.1128/MCB.22.16.5662-5668.2002 PMC133976

[b24] Harrison D. A., McCoon P. E., Binari R., Gilman M. & Perrimon N. *Drosophila* unpaired encodes a secreted protein that activates the JAK signaling pathway. Genes Dev 12, 3252–3263 (1998). 9784499 10.1101/gad.12.20.3252 PMC317220

[b25] Chen H. W. . mom identifies a receptor for the *Drosophila* JAK/STAT signal transduction pathway and encodes a protein distantly related to the mammalian cytokine receptor family. Genes Dev 16, 388–398 (2002). 11825879 10.1101/gad.955202 PMC155335

[b26] Binari R. & Perrimon N. Stripe-specific regulation of pair-rule genes by hopscotch, a putative Jak family tyrosine kinase in *Drosophila*. Genes Dev 8, 300–312 (1994). 8314084 10.1101/gad.8.3.300

[b27] Hou X. S., Melnick M. B. & Perrimon N. Marelle acts downstream of the *Drosophila* HOP/JAK kinase and encodes a protein similar to the mammalian STATs. Cell 84, 411–419 (1996). 8608595 10.1016/s0092-8674(00)81286-6

[b28] Yan R., Small S., Desplan C., Dearolf C. R. & Darnell J. E. Jr. Identification of a Stat gene that functions in *Drosophila* development. Cell 84, 421–430 (1996). 8608596 10.1016/s0092-8674(00)81287-8

[b29] Stec W., Vidal O. & Zeidler M. P. *Drosophila* SOCS36E negatively regulates JAK/STAT pathway signaling via two separable mechanisms. Mol Biol Cell 24, 3000–3009 (2013). 23885117 10.1091/mbc.E13-05-0275 PMC3771960

[b30] Hombria J. C. & Sotillos S. JAK/STAT signalling: STAT cannot play with Ken and Barbie. Curr Biol 16, R98–100 (2006). 16461274 10.1016/j.cub.2006.01.021

[b31] Betz A., Lampen N., Martinek S., Young M. W. & Darnell J. E. Jr. A *Drosophila* PIAS homologue negatively regulates stat92E. Proc Natl Acad Sci USA 98, 9563–9568 (2001). 11504941 10.1073/pnas.171302098 PMC55492

[b32] Kwon S. Y. . The nucleosome remodeling factor (NURF) regulates genes involved in *Drosophila* innate immunity. Dev Biol 316, 538–547 (2008). 18334252 10.1016/j.ydbio.2008.01.033

[b33] Perry S. J. & Lefkowitz R. J. Arresting developments in heptahelical receptor signaling and regulation. Trends Cell Biol 12, 130–138 (2002). 11859025 10.1016/s0962-8924(01)02239-5

[b34] Marullo S. & Coureuil M. Arrestins in host-pathogen interactions. Handb Exp Pharmacol 219, 361–374 (2014). 24292839 10.1007/978-3-642-41199-1_18 PMC5055581

[b35] Mo W. . Nuclear beta-arrestin1 functions as a scaffold for the dephosphorylation of STAT1 and moderates the antiviral activity of IFN-gamma. Mol Cell 31, 695–707 (2008). 18775329 10.1016/j.molcel.2008.06.017

[b36] Bourdeau A., Dube N. & Tremblay M. L. Cytoplasmic protein tyrosine phosphatases, regulation and function: the roles of PTP1B and TC-PTP. Curr Opin Cell Biol 17, 203–209 (2005). 15780598 10.1016/j.ceb.2005.02.001

[b37] Wang Y. . GdX/UBL4A specifically stabilizes the TC45/STAT3 association and promotes dephosphorylation of STAT3 to repress tumorigenesis. Mol Cell 53, 752–765 (2014). 24530303 10.1016/j.molcel.2014.01.020

[b38] Okugawa S. . The SOCS and STAT from JAK/STAT signaling pathway of kuruma shrimp *Marsupenaeus japonicus*: molecular cloning, characterization and expression analysis. Mol Cell Probes 27, 6–14 (2013). 22921512 10.1016/j.mcp.2012.08.003

[b39] Wen R., Li F., Li S. & Xiang J. Function of shrimp STAT during WSSV infection. Fish Shellfish Immunol 38, 354–360 (2014). 24727196 10.1016/j.fsi.2014.04.002

[b40] Sun J. J. . beta-Arrestins Negatively Regulate the Toll Pathway in Shrimp by Preventing Dorsal Translocation and Inhibiting Dorsal Transcriptional Activity. J Biol Chem 291, 7488–7504 (2016). 26846853 10.1074/jbc.M115.698134 PMC4817179

[b41] Sun J. J., Lan J. F., Xu J. D., Niu G. J. & Wang J. X. Suppressor of cytokine signaling 2 (SOCS2) negatively regulates the expression of antimicrobial peptides by affecting the Stat transcriptional activity in shrimp *Marsupenaeus japonicus*. Fish Shellfish Immunol 56, 473–482 (2016). 27492125 10.1016/j.fsi.2016.07.037

[b42] Yan M. . Identification of a JAK/STAT pathway receptor domeless from Pacific white shrimp *Litopenaeus vannamei*. Fish Shellfish Immunol 44, 26–32 (2015). 25659232 10.1016/j.fsi.2015.01.023

[b43] Song X. . A Janus Kinase in the JAK/STAT signaling pathway from *Litopenaeus vannamei* is involved in antiviral immune response. Fish Shellfish Immunol 44, 662–673 (2015). 25839969 10.1016/j.fsi.2015.03.031

[b44] Talati P. G. . Jak2-Stat5a/b Signaling Induces Epithelial-to-Mesenchymal Transition and Stem-Like Cell Properties in Prostate Cancer. Am J Pathol 185, 2505–2522 (2015). 26362718 10.1016/j.ajpath.2015.04.026 PMC4597281

[b45] Bunting K. D. STAT5 signaling in normal and pathologic hematopoiesis. Front Biosci 12, 2807–2820 (2007). 17485261 10.2741/2274

[b46] Peng H. Y. . IL-8 induces miR-424-5p expression and modulates SOCS2/STAT5 signaling pathway in oral squamous cell carcinoma. Mol Oncol (2016). 10.1016/j.molonc.2016.03.001 PMC5423170 27038552

[b47] Nishiyama-Fujita Y., Shimizu T., Sagawa M., Uchida H. & Kizaki M. The role of TC-PTP (PTPN2) in modulating sensitivity to imatinib and interferon-alpha in CML cell line, KT-1 cells. Leuk Res 37, 1150–1155 (2013). 23759247 10.1016/j.leukres.2013.05.008

[b48] Johnson K. J. . PTP1B suppresses prolactin activation of Stat5 in breast cancer cells. Am J Pathol 177, 2971–2983 (2010). 20952588 10.2353/ajpath.2010.090399 PMC2993292

[b49] Kang J. . A nuclear function of beta-arrestin1 in GPCR signaling: regulation of histone acetylation and gene transcription. Cell 123, 833–847 (2005). 16325578 10.1016/j.cell.2005.09.011

[b50] Livak K. J. & Schmittgen T. D. Analysis of relative gene expression data using real-time quantitative PCR and the 2(-Delta Delta C(T)) Method. Methods 25, 402–408 (2001). 11846609 10.1006/meth.2001.1262

[b51] Du X. J., Zhao X. F. & Wang J. X. Molecular cloning and characterization of a lipopolysaccharide and beta-1,3-glucan binding protein from fleshy prawn (*Fenneropenaeus chinensis*). Mol Immunol 44, 1085–1094 (2007). 16930711 10.1016/j.molimm.2006.07.288

[b52] Wang S., Liu N., Chen A. J., Zhao X. F. & Wang J. X. TRBP homolog interacts with eukaryotic initiation factor 6 (eIF6) in *Fenneropenaeus chinensis*. J Immunol 182, 5250–5258 (2009). 19380771 10.4049/jimmunol.0802970

[b53] Jiang H. S. . A new group of anti-lipopolysaccharide factors from Marsupenaeus japonicus functions in antibacterial response. Developmental and comparative immunology 48, 33–42 (2015). 25218642 10.1016/j.dci.2014.09.001

